# *Bryophyllum pinnatum* Compounds Inhibit Oxytocin-Induced Signaling Pathways in Human Myometrial Cells

**DOI:** 10.3389/fphar.2021.632986

**Published:** 2021-02-18

**Authors:** Stefanie Santos, Leonie Zurfluh, Mónica Mennet, Olivier Potterat, Ursula von Mandach, Matthias Hamburger, Ana Paula Simões-Wüst

**Affiliations:** ^1^ Department of Obstetrics, University Hospital Zurich (USZ), University of Zurich (UZH), Zurich, Switzerland; ^2^ Division of Pharmaceutical Biology, University of Basel, Basel, Switzerland; ^3^ Weleda AG, Arlesheim, Switzerland

**Keywords:** *Bryophyllum pinnatum*, *Kalanchoe pinnata*, myometrium cells, oxytocin, MAPK, intracellular calcium, cell signaling

## Abstract

*Bryophyllum pinnatum* has been used in the treatment of premature labor, first in anthroposophic hospitals and, recently, in conventional settings as an add-on medication. *In vitro* work with hTERT human myometrial cells showed that *B. pinnatum* leaf press juice inhibits the increase of intracellular free calcium concentration induced by oxytocin, a hormone known to play a role in labor. Our aim was to identify fractions/compounds in *B. pinnatum* press juice that contribute to this inhibitory effect, and to investigate their effect on oxytocin-driven activation of the MAPK cascade. Several fractions/compounds from *B. pinnatum* press juice led to a concentration-dependent decrease of oxytocin-induced increase of intracellular free calcium concentration, but none of them was as strong as *B. pinnatum* press juice. However, the combination of a bufadienolide and a flavonoid-enriched fraction was as effective as *B. pinnatum* press juice, and their combination had a synergistic effect. *B. pinnatum* press juice inhibited oxytocin-driven activation of MAPKs SAPK/JNK and ERK1/2, an effect also exerted by the bufadienolide-enriched fraction. The effect of *B. pinnatum* press juice on oxytocin-induced signaling pathways was comparable to that of the oxytocin-receptor antagonist and tocolytic agent atosiban. Our findings further substantiate the use of *B. pinnatum* press juice preparations in the treatment of preterm labor.

## Introduction

Every year, around 15 million neonates worldwide are born too early. Prematurity, (i.e. birth before 37 weeks of pregnancy) is the number one cause of neonatal deaths, and the leading cause of death in children under 5 years of age. A number of pharmacological agents known as tocolytics—including beta-sympathomimetic drugs, oxytocin receptor (OTR) antagonists, and calcium channel inhibitors—have been introduced for inhibiting preterm uterine contractions that are responsible for a considerable part of preterm births (Romero et al., 2014). Tocolysis is usually performed for 48 h to allow corticosteroid administration to the mother in order to achieve fetal lung maturation, and *in utero* transfer to a perinatal center (Romero et al., 2014). However, to date there are no fully satisfactory tocolytics as often birth still occurs prematurely. Moreover, treatment with the usual tocolytic agents is often accompanied by various, in part serious side-effects. These include tachycardia, dyspnea, palpitation, pulmonary edema, and hyperglycemia [in the case of the sympathomimetic drugs (Arrowsmith et al., 2010)], nausea, dizziness, headache, and tachycardia [OTR antagonists (Flenady et al., 2014a)] and flushing, hypotension, and suppression of heart rate [calcium channel blockers (Flenady et al., 2014b)].

Labor is clinically manifested by rhythmic uterine contractions leading to the expulsion of the baby. Oxytocin (OT) is a potent physiological stimulator of myometrial contractions, and its receptor and the downstream signaling pathways are attractive targets for drug development aimed at managing preterm labor (Arrowsmith et al., 2010). At the cellular level, the mechanism by which OT leads to stimulation of the uterus is very complex. Binding of OT to its receptor (OTR) leads to OTR coupling with G proteins promoting myometrial contractions through multiple signaling pathways (Arrowsmith and Wray, 2014; Kim et al., 2017). Gαq/11-mediated signaling activates phospholipase C-β, which in turn hydrolyses phosphatidylinositol 4,5-bisphosphate into inositol 1,4,5-trisphosphate (IP_3_) and diacylglycerol. IP_3_ leads to release of calcium ions (Ca^2+^) from the endoplasmic reticulum into the cytoplasm, and diacylglycerol activates protein kinase type C. A high Ca^2+^-concentration in the cytoplasm promotes myometrial contraction through activation of myosin light chain kinase, known as canonical pathway. Protein kinase type C activates the mitogen-activated protein kinase (MAPK) cascade (Arrowsmith and Wray, 2014; Kim et al., 2017), resulting in increased phospholipase A_2_ activity and prostaglandin E_2_ production which also contributes to contraction. In recent years, the importance of inflammatory processes in labor at term and preterm became apparent (Kim et al., 2015). The main MAPKs are the extracellular signal-regulated kinases (ERKs) 1 and 2 (ERK1/2), stress-activated protein kinase or c-Jun amino-terminal kinases (SAPK/JNK) and p38 (Roux and Blenis, 2004; Cuenda and Rousseau, 2007). Due to the direct impact of OT on myometrium contractility, OTRs are attractive targets. A widely used competitive inhibitor of the OTR is atosiban (Flenady et al., 2014a).

*Bryophyllum pinnatum* (Lam.) Oken, [syn. *Kalanchoe pinnata* (Lam.), Pers.; family *Crassulaceae*] has been widely used in traditional medicine of tropical countries, in the treatment of wounds, diabetes mellitus, joint pain, headache, etc. [see (Fürer et al., 2016) and references therein]. In 1970, *B. pinnatum* was introduced in obstetrics at the anthroposophic Herdecke Community Hospital (Germany) for the treatment of preterm labor. In Switzerland, products containing press juice of *B. pinnatum* leaves are nowadays prescribed for the same indication (Fürer et al., 2016; Schenkel et al., 2018). Several clinical studies have shown a very good tolerability of *B. pinnatum* preparations for this (Plangger et al., 2006; Fürer et al., 2015b; Simões-Wüst et al., 2018) and other indications (Betschart et al., 2013; Lambrigger-Steiner et al., 2014). *In vitro* studies showed that *B. pinnatum* reduces the strength of human myometrium contractions (Santos et al., 2019a; Santos et al., 2019b). In human myometrial cells (hTERT-C3 cell line), leaf press juice of *B. pinnatum* (BPJ) lowered the OT-induced increase of intracellular calcium concentrations [(Ca^2+^)_i_] (Simões-Wüst et al., 2010).

Previous phytochemical studies on *B. pinnatum* showed that flavonoid glycosides and bufadienolides are the major classes of secondary metabolites in leaves (Fürer et al., 2013; Oufir et al., 2015). The presence of flavonoid glycosides (derivatives of quercetin, myricetin, diosmetin, and kaempferol) and bufadienolides [bersaldegenin-1-acetete, bryophyllin A, bersaldegenin-3-acetate, and bersaldegenin-1,3,5-orthoacetate (BO)] was shown.

An efficient tocolysis in preterm labor remains challenging and therefore the investigation of alternatives to standard therapy is of continued importance. We here investigated the inhibitory effects of *B. pinnatum* press juice compounds on OT-induced intracellular signaling that plays a major role in the physiology of uterine contractions. Specifically, we have compared the effects on intracellular calcium levels and activation of MAPK proteins with those of the OTR-antagonist and tocolytic agent atosiban.

## Materials and Methods

### Cell Culture

Human myometrial telomerase reverse transcriptase cells (hTERT-C3) (Condon et al., 2002; Devost and Zingg, 2007) provided by M. Grãos (Biocant, Cantanhede, Portugal), were cultured in a 1:1 mixture of DMEM and F-12 supplemented with antibiotics (100 U/mL penicillin and 100 μg/ml streptomycin) and 10% (v/v) heat-inactivated fetal bovine serum (FBS) (all from Gibco, Paisley, United Kingdom). Pregnant human myometrial cells (PHM1-41, ATCC® CRL-3046TM) were maintained in ATCC-formulated DMEM (ATCC® No. 30–2002) supplemented with 10% (v/v) heat inactivated FBS, 2 mM glutamine (Gibco, Paisley, United Kingdom), and 0.1 mg/mL G-418 (Carl Roth, Zurich, Switzerland).

### Plant Material

Plant material of *B. pinnatum* originated from two different harvests. Weleda Brazil provided leaves harvested in S. Roque, Brazil, on March 25, 2014. A voucher specimen (ZSS 29717) was deposited at The Zurich Succulent Plant collection. Immediately after the collection, leaves were sent by airmail in a refrigerated container to Weleda AG, Arlesheim, Switzerland. In addition, the Weleda AG branch located in Schwäbisch Gmünd, Germany, provided leaves harvested in July and August 2010. A voucher specimen (ZSS 29715) was deposited at the Zurich Succulent Plant collection.

Plant material of *B. daigremontianum* was provided by the Ita Wegman Hospital Arlesheim, Switzerland, in September 2010. A voucher specimen (No. 838) was deposited at the Division of Pharmaceutical Biology, University of Basel, Switzerland.

### *Bryophyllum pinnatum* Leaf Press Juice (BPJ)

BPJ was prepared from leaves harvested in S. Roque, Brazil. The press juice was obtained by mechanical pressing of leaves in a roller mill. The procedure was identical to the initial steps of the protocol used for the production of the active ingredient of Weleda Bryophyllum 50% chewing tablets (Weleda AG, Arlesheim, Switzerland). The amount of bufadienolides and flavonoids in BPJ was 0.012 and 0.072 mg/ml (based on the determined content of the flavonoid aglycones—0.034 mg/ml), respectively (Oufir et al., 2015; Santos et al., 2019a). The suspension was filtered using a 150 mm diameter paper filter (Schleicher and Schuell, Dassel, Germany), and aliquots were kept at −80°C until use.

### Bufadienolide and Flavonoid-Enriched Fractions (BEF and FEF)

Enriched fractions originated from earlier studies (Fürer et al., 2015a; Bachmann et al., 2017). Frozen fresh leaves (Weleda, Schwäbisch Gmünd, Germany) were lyophilized, powdered in a mortar, and extracted with MeOH. The MeOH extract was partitioned between H_2_O and CH_2_Cl_2_. The aqueous phase was further fractionated by column chromatography (with Diaion HP20 resin) and, after a first elution with H_2_O to remove the highly polar compounds, FEF was obtained by elution with MeOH. Evaporation of the CH_2_Cl_2_-soluble phase yielded a residue (Fürer et al., 2015a) that was further purified to afford BEF (Bachmann et al., 2017). The amount of flavonoids in FEF was estimated to be approx. 8.28% based on the determined content of the flavonoid aglycones (4.14%) while the amount of bufadienolides in BEF was found to be 9.10% (Bachmann et al., 2017). BEF and FEF stock solutions (1.3 and 10.0 mg/ml, respectively) were prepared in DMSO.

### Flavonoid Aglycon Mix (A-Mix)

After hydrolysis of FEF, a content of 4.1% of total flavonoid aglycons was determined, and relative proportions were 74.6% of quercetin, 16.7% of myricetin, 4.6% of diosmetin, and 4.0% of kaempferol (Bachmann et al., 2017). A mixture of the four aglycons in these proportions (A-Mix) was prepared in DMSO at a concentration of 0.4 mg/ml.

### Bersaldegenin-1,3,5-orthoacetate (BO)

The compound was previously isolated from *B. daigremontianum* (Fürer et al., 2013). The amount of BO in BPJ was 0.002 mg/ml (Oufir et al., 2015). A stock of 0.023 mg/ml was prepared in DMSO.

### Drugs, Reagents and Test Substances

OT and Digitonin were obtained from Sigma-Aldrich (St. Louis, USA). Fura-2 and Pluronic F-127 were purchased from Molecular Probes-Invitrogen (California, USA). Atosiban (Tractocile®, 7.5 mg/ml injectable solution) was purchased from Ferring Pharmaceuticals (Baar, Switzerland) and dimethyl sulfoxide (DMSO) from Sigma (France).

All substances tested were diluted in Krebs solution or cell media prior to experiments being performed. The DMSO concentration in test substances was adjusted to 0.1% in calcium experiments and 0.2% in phosphorylation experiments. Control wells were treated to contain the same concentration of DMSO.

### Measurement of Intracellular Calcium Levels

hTERT-C3 (8 × 10^4^ cell/mL) and PHM1-41 (10 × 10^4^ cell/mL) cells were seeded into 96-well black microplates (Corning Inc., USA) two days before experiments were performed. Measurement of intracellular calcium levels was performed as previously described with some adaptations (Simões-Wüst et al., 2010). Briefly, cells were loaded with 10 µM Fura-2/AM reconstituted in DMSO as a 1 mM stock solution and 0.06% (w/v) Pluronic F-127 in fresh medium. After 1 h incubation at 37°C, Fura-2 was replaced by fresh medium and cells were incubated for 30 min. Thereafter, cells were washed twice with 100 µL sodium salt solution (140 mM NaCl, 5 mM KCl, 1 mM CaCl_2_, 1 mM MgCl_2_, 10 mM Glucose, 10 mM HEPES-Na^+^, pH = 7.4). Test substances were added in fresh sodium salt solution and fluorescence was read for 4 min, followed by stimulation with 100 nM OT (4 min reading). At the end of the experiments, cells were permeabilized with 200 µM digitonin followed by Tris-EGTA solution (Tris 1M, EGTA 200 mM, pH = 10.2). Fluorescence was read for 2 min. For each substance tested, 4 (PHM1-41 cell line) to 6 (hTERT-C3 cell line) independent cultures, carried out in quadruplicate, were used. A negative control, with no test substance added (or DMSO) to the culture medium and a positive control (5,000 nM atosiban) were included in each plate.

Fluorescence was measured at emission of 510 nm by illuminating the cells with an alternating 340/380 nm light every 4 s, using a microplate fluorescence reader (EnVision Multilabel Reader, Perkin Elmer). Fluorescence intensities were acquired using the Wallac EnVision Manager software. The relative fluorescence units readings were converted to [Ca^2+^]_i_ values (in nM) using the following formula:

$${\left[Ca^{2+}\right]}_{i}=K_{d}\times Q\left(R-R_{\text{min}}\right)/\left(R_{max}-R^{\prime }\right).$$

where K_d_ = dissociation constant of the Ca^2+^/Fura-2 complex (224 nM); Q = F_min_/F_max_ at 380 nm (F_max_ after digitonin and F_min_ after EGTA); R = F_340_ nm/F_380_ nm (F—fluorescence intensity); R_max_ at maximum Ca^2+^ concentration (after digitonin) and R_min_ at minimum Ca^2+^ concentration (after Tris-EGTA).

The variation of intracellular Ca^2+^ concentration (Δ[Ca^2+^]_i_) in each well was calculated by subtracting basal readings from the peak of [Ca^2+^]_i_ after stimulation with OT (5 highest points). The Δ[Ca^2+^]_i_ was normalized to control values.

To characterize the effect of combinations, the median-effect method was used (Chou, 2006). This method is based on the mass action law and allows a quantitative definition of the interaction between two different drugs. The combination Index (CI) is widely used to asses both beneficial and adverse interactions between pharmaceuticals. CI quantitatively determines/simulates a measure of the extent of drug combination at all doses and all effects with small number of data points. CI is calculated according to which the interaction between two drugs can be classified as antagonistic (CI > 1), additive (CI = 1) or synergistic (CI < 1). CI values were calculated using Compusyn software.

### Phosphorylation Experiments

hTERT-C3 cells (4.7 × 10^4^ cell/mL) were seeded into 6-well plates three to 4 days before experiments were performed. Once 90% confluence was reached, cells were treated with OT (100 nM) for 2, 5, 15, 30 or 45 min. To investigate the modulation of OT-induced phosphorylation under these conditions, cells were pre-treated with BPJ (2% corresponding to 20 mg/mL), atosiban (100 nM) or just medium. In additional experiments, cells were pretreated with BPJ (20 mg/mL), BEF (2.20 μg/mL), FEF (17.39 μg/mL), A-Mix (0.68 μg/mL), BEF plus FEF and BEF plus A-Mix (same concentrations as in the single treatments), or just medium for 30 min before stimulation with OT for 5 min.

Proteins (20 µg) were separated in a 12% SDS polyacrylamide gel, and transferred to a PVDF membrane. When protein samples from one experiment were run in more than one gel, a normalisation sample (NS) constituted by proteins extracted from hTERT-C3 cells exposed to OT for 5 min was used in all gels to decrease variability between blots. Membranes were incubated in primary antibody: GAPDH (1:2000), phospho-p38 (Thr180/Tyr182; 1:1,000), phospho-p44/42 ERK1/2 (Thr202/Tyr204; 1:1,000), phospho-SAPK/JNK (Thr183/Tyr185; 1:1,000) overnight at 4°C and in the appropriated Horseradish peroxidase (HRP)-conjugated secondary antibody (all from Cell Signalling, Allschwil, Switzerland) for 1 h at room temperature the next day. Equal loading was confirmed by blotting the membranes for the house-keeping gene GAPDH. Signal detection was done using SupersignalTM West Pico Plus Chemiluminescent Substrate (Thermo scientific, Rockford, USA). Detection and quantification of band intensities was performed using FusionCapt Advance system (Vilber, Eberhardzell, Germany). For each substance tested, four independent cultures of hTERT-C3 cell line were used.

### Statistical Analysis

All results were expressed as mean ± standard error of the mean (SEM) and statistical analyses were performed with Graphpad Prism software. The Shapiro-Wilk test was used to check normal distribution. For intracellular calcium measurements one-way ANOVA with Dunnett’s post-hoc test was conducted to compare different concentrations of each test substance to the corresponding control. Ordinary two-way ANOVA was used to compare different dose-dependency treatments. For phosphorylation study, repeated measures two-way ANOVA with Dunnett’s multiple comparison was used to evaluate differences between each time point to time 0 min, and to OT-treated. To compare the different test substances to non-stimulated control or to OT-stimulated cells, the Mann-Whitney was used. Values were considered to be statistically significant if *p* < 0.05.

## Results

### Inhibition of OT-induced rise of [Ca^2+^]_i_ by BPJ fractions/compounds

**FIGURE 1:**
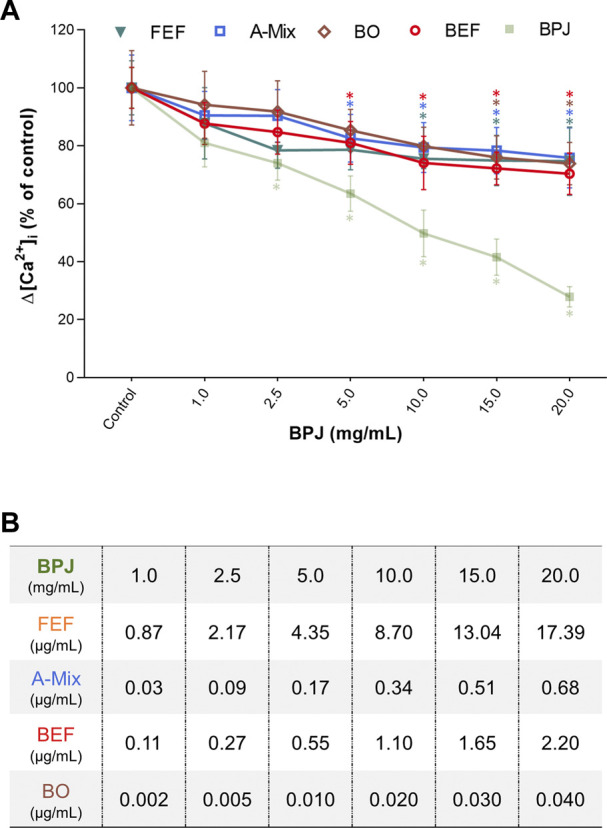
Concentration-dependent effects of BPJ fractions/compounds on the OT-induced increase of [Ca^2+^]_i_. Myometrial hTERT-C3 cells were pre-treated with BPJ, FEF, A-Mix, BEF or BO prior to stimulation with 100 nM of OT **(A)**. Values represent the mean ± SEM of six independent experiments performed in quadruplicate and are expressed as percentage of control; *p < 0.05. In **(B)**, the concentrations of FEF, BEF, A-Mix and BO corresponding to the BPJ concentrations are shown.

We previously showed that BPJ inhibits OT-induced rise of [Ca^2+^]_i_ (Simões-Wüst et al., 2010). To investigate the contribution of different constituents/fractions of *B. pinnatum* on the OT-induced rise of [Ca^2+^]_i_, hTERT-C3 cells were pre-incubated with FEF, BEF, A-Mix and BO. The Δ[Ca^2+^]_i_ decreased progressively and in a statistically significant way when cells were pre-incubated with FEF and A-Mix (FEF: *p* = 0.043, A-Mix: *p* = 0.0008). Compared to control, the values of Δ[Ca^2+^]_i_ were significantly lower at 4.35 μg/ml of FEF (*p* = 0.030) and 0.17 μg/ml of A-Mix (*p* = 0.014; Figure 1A).

As shown in Figure 1A, the effect of BEF and BO on the OT-induced rise of [Ca^2+^]_i_ was concentration dependent (BEF: *p* = 0.0001, BO: *p* = 0.022). When each concentration was compared to control, a significant difference was obtained with 0.55 μg/ml of BEF (*p* = 0.01) and 0.035 μg/ml of BO (*p* = 0.028). Under our experimental conditions, none of the fractions/compounds had an effect that was equally strong as that of BPJ (Figures 1A,B). Concentrations of FEF, BEF, A-Mix and BO used during the experiments are correspondent to those found in BPJ (Figure 1B).

### Inhibition of OT-Induced Rise of [Ca^2+^]_i_ by Combinations of BEF With FEF, and of BEF With A-Mix

**FIGURE 2:**
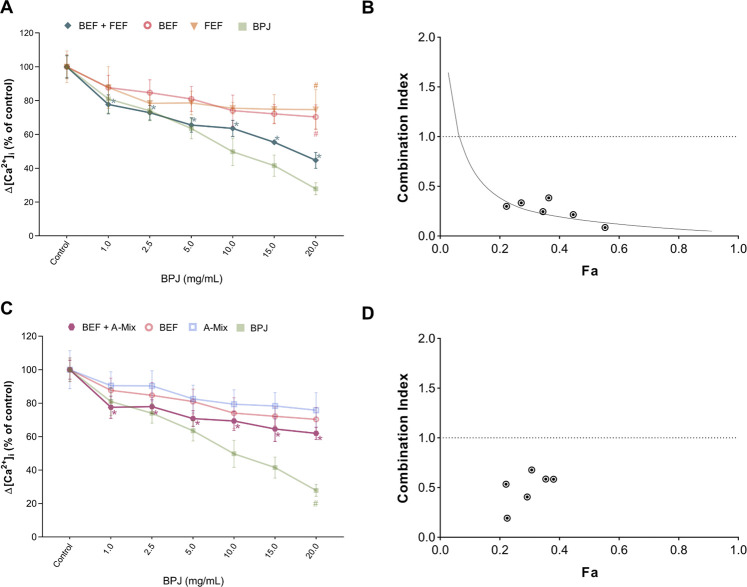
Effect of the combination of BPJ fractions/compounds on the OT-induced rise of [Ca^2+^]_i_. Cells were pre-treated with BEF plus FEF **(A)**, or with BEF plus A-Mix **(C)** prior to stimulation with 100 nM OT. Results of pre-treatment with BPJ and each substance alone are shown in transparent lines (significance symbols regarding comparison to control omitted). Values represent the mean ± SEM of six independent experiments performed in quadruplicate and are expressed as percentage of control; *p < 0.05 compared with control; ^#^ *p* < 0.05 compared to combination. Combination index (CI) values of the combination of BEF with FEF **(B)** or with A-Mix **(C)** were calculated from the concentration-response curves. Data were analyzed by the median-effect method. Fa: fraction affected.

To assess whether more than one fraction/compound mixture was needed to obtain an effect comparable to that of BPJ, combinations of BEF with FEF, and of BEF with A-Mix were investigated, again at test concentrations that corresponded to their content in BPJ. BEF plus FEF led to a concentration-dependent inhibition of Δ[Ca^2+^]_i_, whereby all concentrations were significantly different from control (*p* < 0.0001). No statistically significant difference was observed when comparing the effect of BEF plus FEF with that of BPJ. The combination of the highest concentrations of BEF and FEF (2.20 and 17.39 μg/ml, respectively) was significantly different from each substance alone at the same concentrations (BEF: *p* = 0.030, FEF: *p* = 0.009; Figure 2A).

Pre-incubation of cells with the combination of BEF plus A-Mix also showed a concentration-dependent effect (*p* < 0.0001) on the OT-induced rise of [Ca^2+^]_i_, and at all test concentrations the effect was significantly different from control. However, the effect of the combination was generally weaker than that of BPJ. The combination of the highest concentrations of BEF and A-Mix (2.20 and 0.68 μg/ml, respectively) was significantly different from BPJ (*p* = 0.0035; Figure 2C).

### Characterization of the Combined Effects

The median-effect method was used to analyze the data for antagonism, additivity or synergism of the combinations. Figures 2B,D show that combination index (CI) values less than one were obtained with the combinations studied, which is indicative for synergistic interaction. Fraction affected (Fa) values for the various combinations of BEF and FEF ranged between 0.2 and 0.6, indicating that the synergistic interaction was observed when the inhibition of OT-induced increase of intracellular Ca^2+^ levels was 20–60% (Figure 2B). Fa values for the combination of BEF and A-Mix ranged between 0.2 and 0.4, reflecting a weaker maximal inhibition by this combination (Figure 2D).

### BPJ inhibits OT-induced rise of [Ca^2+^]_i_ in PHM1-41 myometrial cells

**FIGURE 3:**
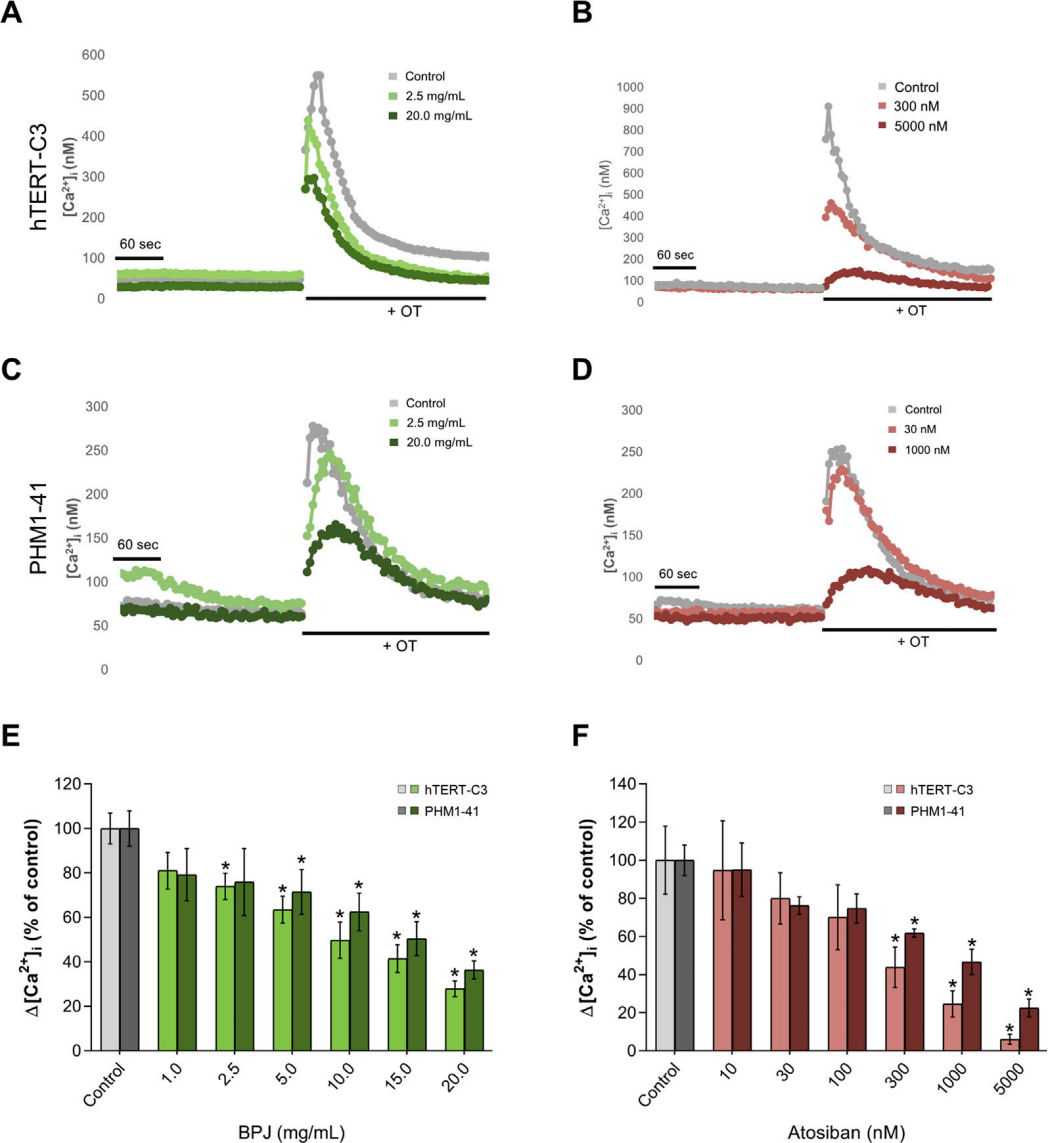
Comparison between the effects of BPJ and atosiban on OT-induced rise of [Ca^2+^]_i_ in myometrial cell lines. Time-course of OT-induced [Ca^2+^]_i_ response in hTERT-C3 **(A, B)** and PHM1-41 **(C, D)** cells when pre-incubated in the absence (a-d, grey lines) or in the presence of BPJ **(A, C)** or atosiban **(B, D)** [Ca^2+^]_i_ was measured for 4 min before stimulation with 100 nM OT. Data shown are from one representative experiment. Concentration-dependent effect of BPJ **(E)** or atosiban **(F)** on the oxytocin-induced [Ca^2+^]_i_ increase in hTERT-C3 (lighter color) and PHM1-41 (darker color) cells. Values represent themean ± SEM of 4 (PHM1-41) or 6 (hTERT-C3) independent experiments performed in quadruplicate and are expressed as percentage of control; *p < 0.05.

As previously shown, the exposure of hTERT-C3 cells to 100 nM OT induced an increase of [Ca^2+^]_i_ with a peak response at about 10–20 s after stimulation, and a subsequent decrease to resting conditions (Figures 3A,B). Pre-incubation with BPJ (0.1%–2.0% corresponding to 1.0–20.0 mg/mL) led to a concentration dependent decrease of the [Ca^2+^]_i_ peak induced by OT (*p* < 0.0001; Figure 3A). To verify if this effect was cell line dependent, experiments were conducted in a second myometrial cell line (PHM1-41). A peak response of [Ca^2+^]_i_ was observed at 12–20 s after stimulation with OT (Figures 3C,D). When pre-incubated with BPJ, a decrease of the OT induced increase of cytosolic [Ca^2+^]_i_ peak was observed (Figure 3C). BPJ thus promoted a concentration-dependent effect on ▵[Ca^2+^]_i_ in both cell lines (*p* < 0.0001; Figure 3E), with significant effects at concentrations >2.5 mg/mL in hTERT-C3 cells (*p* = 0.012), and >5.0 mg/mL in PHM1-41 cells (*p* = 0.026).

In both cell lines, the effect of BPJ was compared with that of the OTR antagonist atosiban. Pre-incubation of hTERT-C3 and PHM1-41 with atosiban led to a decrease of the OT-induced [Ca^2+^]_i_ peak (Figures 3B,D, respectively), and promoted a concentration-dependent effect on Δ[Ca^2+^]_i_ (*p* < 0.0001, both cell lines; Figure 3F). The highest concentration of atosiban (5000 nM) lowered [Ca^2+^]_i_ to 6.04 and 22.46% of control in hTERT-C3 and PHM1-41 cells, respectively.

### BPJ Inhibits OT-Induced Phosphorylation of MAPK Proteins

**FIGURE 4:**
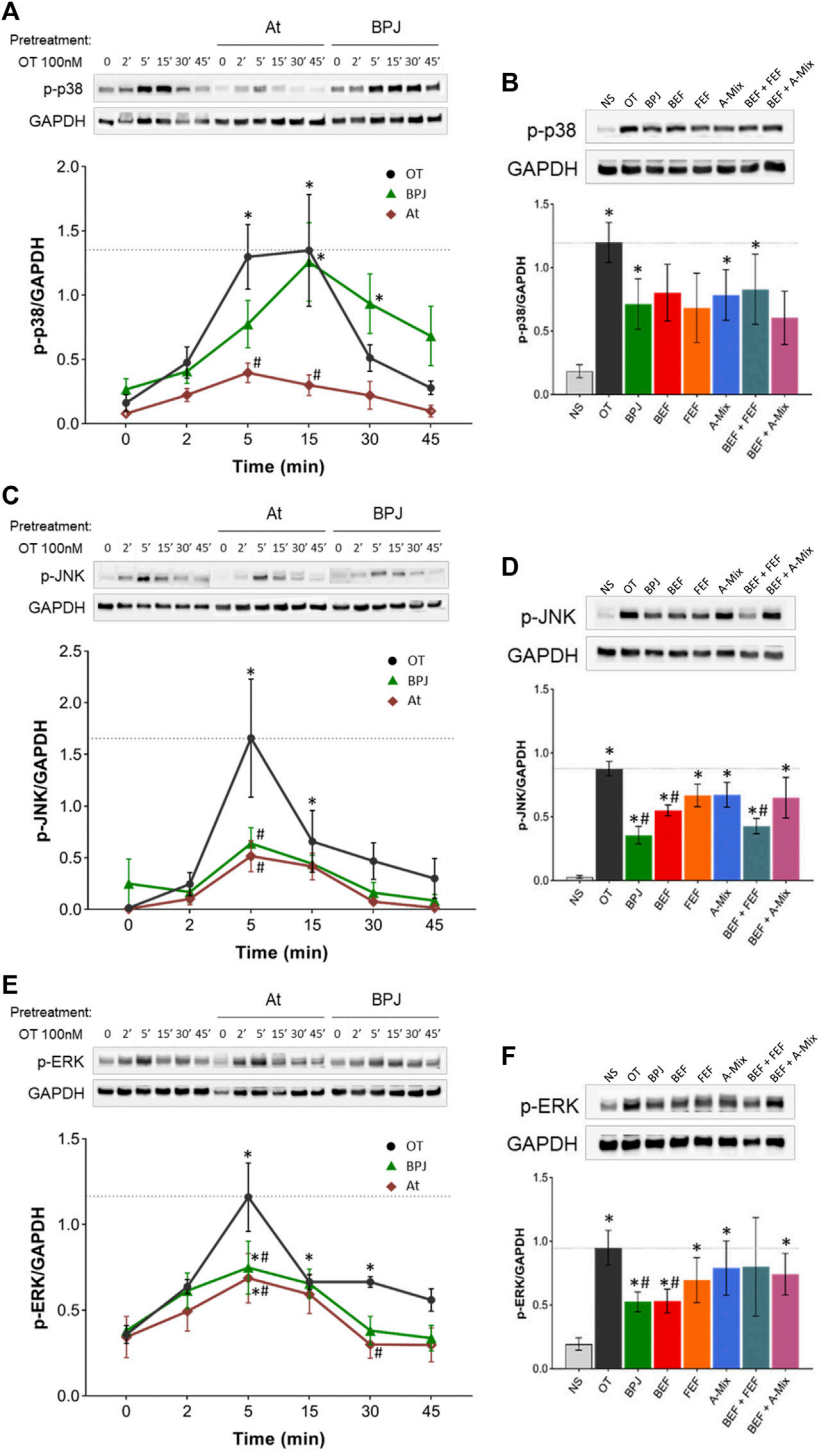
Effect of BPJ fractions/compounds and atosiban on OT-induced MAPKs phosphorylation. In the time-course experiments, hTERT-C3 cells were pre-treated with or without 20 mg/mL BPJ or 100 nM atosiban for 30 min, before incubation with 100 nM OT for 2, 5, 15, 30, and 45 min **(A, C, E)**. To compare the effects of BPJ and the various fractions/compounds, cells were pre-treated with 20 mg/mL BPJ, 2.20 μg/mL BEF, 17.39 μg/mL FEF, 0.68 μg/mL A-Mix, BEF plus FEF, or BEF plus A-Mix (same concentrations as for single fractions) for 30 min before stimulation with OT for 5 min **(B, D, F)**. Whole cell proteins were subjected to western blot analysis with antibodies against phosphorylated p38 (A, B), SAPK/JNK (C, D) and ERK1/2 **(E, F)**; matching densitometry analyses are depicted bellow the representative blots. Samples from the same experiment were processed in parallel and membranes were probed with GAPDH to confirm equal loading. Blot images are from a representative experiment, and line between bands delineate boundary between the gels that were cropped. Values represent the mean ± SEM of four independent experiments; *p < 0.05 compared with control; ^#^ *p* < 0.05 compared to OT-treated.

We investigated the effect of BPJ on OT-induced phosphorylation of p38, SAPK/JNK and ERK1/2 in hTERT-C3 cells. Time-course experiments revealed that the amounts of the phosphorylated forms of these three MAPKs increased markedly during the first 5 min of incubation with OT (Figures 4A,C,E). The amounts of phosphorylated p38 (p-p38) remained constant for further 10 min before starting to decrease (Figure 4A), whereas phosphorylated forms of SAPK/JNK (*p*-SAPK/JNK) and ERK1/2 (*p*-ERK1/2) decreased already after 10 min of incubation with OT (Figures 4C,E). After 45 min, the levels of p-p38, *p*-SAPK/JNK and *p*-ERK1/2 were comparable to basal values. Data are expressed relatively to the house-keeping protein GAPDH; due to the short incubation times, increased amounts of the phosphorylated forms are likely to reflect direct effects on phosphorylation.

As shown in Figure 4A, pre-treatment with BPJ delayed, but did not prevent, OT-triggered increase in p-p38. BPJ seemed to attenuate the effect of OT on the amount of p-p38 at 5 min, but the difference did not reach statistical significance. For comparison, atosiban was included in the time-course experiments. This OTR antagonist led to a significant inhibition of OT-induced increase in p-p38 at 5 and 15 min (*p* = 0.001 and *p* = 0.001; Figure 4A). The effects of pre-treating the cells with BPJ fractions/compounds alone or combined on OT-induced increase of p-p38 were comparable to the one of BPJ, i.e., none of the test substances promoted a significant decrease after at 5 min incubation with OT (Figure 4B).

Pre-treatment with BPJ led to a significant attenuation of the maximal OT-driven increase of *p*-SAPK/JNK (*p* = 0.0002). A similar inhibition was observed when cells were pre-incubated with atosiban (*p* = 0.0002; Figure 4C). Pre-treatment with BEF alone and even more BEF plus FEF induced a significant decrease in the maximal OT-induced *p*-SAPK/JNK amount (in both cases *p* = 0.029). The effect of the combination was similar to the one of BPJ (Figure 4D).

Considering *p*-ERK1/2, pre-treatment with BPJ significantly reduced the OT-driven maximal activation, which was observed after 5 min incubation (*p* = 0.006). Also, in this case, atosiban reduced maximal activation (*p* = 0.001; Figure 4E). The results obtained with BPJ fractions/compounds showed that only BEF promoted an inhibition similar to that observed upon pre-treatment with BPJ (*p* = 0.029). None of the other fractions/compounds significantly reduced the OT-driven increase of *p*-ERK1/2 (Figure 4F).

## Discussion

Our results show that BPJ inhibits several OT-induced signaling pathways involved in the regulation of myometrial contractility. Apart from a strong inhibition of the OT-induced increase of [Ca^2+^]_i_ by BPJ, various fractions obtained from BPJ exhibit similar, albeit weaker, effects. In contrast, the combination of BEF and FEF has an effect on the canonical signaling pathway that is comparable to that of BPJ, and the combination of these fractions enriched in bufadienolides (BEF) and flavonoid glycosides (FEF) is synergistic. Another OT-induced pathway, namely the activation of the MAPKs SAPK/JNK and ERK1/2 is inhibited by BPJ, whereby the bufadienolide fraction seems to be chiefly responsible for the inhibition of ERK1/2 phosphorylation, and the combination of BEF plus FEF for the inhibition of SAPK/JNK phosphorylation. In contrast, activation of p38 is hardly affected.

To better understand which compound classes in BPJ might be responsible for its effect on the OT-induced increase of [Ca^2+^]_i_, BEF, FEF and A-Mix were tested at concentrations that corresponded to those in BPJ (Figure 1A). In addition, the effects of BO, a major bufadienolide (Wagner et al., 1986) known to be present in BPJ (Oufir et al., 2015), was tested at a corresponding concentration. Given that bufadienolides and flavonoid glycosides are the two major classes of secondary metabolites in BPJ that may contribute to the inhibition of the OT-induced increase of [Ca^2+^]_i_, we studied the effect of a combination of BEF and FEF (Figure 2). Considering that the intestinal flora hydrolyzes flavonoid glycosides to the corresponding aglycones (Cassidy and Minihane, 2017), we also investigated the combination of BEF with the flavonoid aglycones (A-Mix; Figure 2). The amounts of flavonoid aglycons in BPJ and FEF, of bufadienolides present in BPJ and BEF and of BO in BPJ have been determined previously (Oufir et al., 2015; Bachmann et al., 2017; Santos et al., 2019a). Since even longer incubations at higher concentrations of BPJ fractions/compounds did not affect cell viability (Santos et al., 2019a), it is likely that the results reflect a true effect on OT-signaling. BEF, FEF and A-Mix promoted a concentration-dependent lowering of OT-induced increase of [Ca^2+^]_i_, but these effects were not as strong as that of BPJ (Figure 1). However, the effect of a combination of BEF and FEF—but not of BEF plus A-Mix—was comparable to that of BPJ. This indicates that several compound classes in BPJ contribute to the inhibition of the OT-induced increase of [Ca^2+^]_i_, and that the effects of BEF and FEF (Figure 2A) are synergistic.

Synergistic effects of compounds with different mechanisms of action have often been postulated as being important for the pharmacological activity of phytomedicines (Wagner, 2011; Butterweck and Nahrstedt, 2012). However, our present work is one of only a few cases where this has been shown experimentally. The complex regulation of [Ca^2+^]_i_ and, thus, of myometrial contractility (Arrowsmith and Wray, 2014) offers numerous possible targets for compounds present in BPJ. Synergistic effects could occur by compounds inhibiting 1) OT-binding to OTR, 2) IP_3_-binding to its receptor in the endoplasmic reticulum or 3) phosphorylation by signaling kinases, or even molecules that affect membrane depolarization with concomitant modulation of voltage-gated calcium channels. Inhibition of membrane Na^+^/K^+^-ATP-ases by bufadienolides (Perera Cordova et al., 2016) could indirectly affect the contribution of voltage-dependent calcium channels to the [Ca^2+^]_i_. Results showing that BEF has stronger effects on MAPK activation than FEF indicate that bufadienolides possibly regulate contractility also by this pathway, even though the effect was visible only after a few minutes, i.e. at later time points than the effects on the [Ca^2+^]. Flavonoid glycosides, on the other hand, have been shown to affect several kinase signaling cascades, and to have strong anti-inflammatory effects (Romano et al., 2013). Flavonoids in *B. pinnatum* may thus possibly modulate the inflammation mediated processes involved in the regulation of myometrial cell contractility. Further research is needed to find out with which biomolecules do bufadienolides and flavonoid glycosides interact in human myometrium.

Press juice of *B. pinnatum* leaves (BPJ) is the active ingredient of preparations that are being used in Switzerland to treat preterm labor (Schenkel et al., 2018), often as an add-on treatment (Fürer et al., 2015b). The present work confirms earlier results showing that BPJ inhibits the canonical pathway of OT-induced increase of contractility in hTERT-C3 human myometrium cells (Simões-Wüst et al., 2010). A comparable inhibition of the OT-induced increase of [Ca^2+^]_i_ has now been observed in PHM1-41 myometrial cells (Figure 3E), showing that the effect is not restricted to the (transformed) hTERT-C3 myometrial cells. In both cell lines, pre-treatment with the OT-antagonist atosiban led to an inhibition of the canonical pathway. It is tempting to speculate that BPJ compounds exert effects that are at least in part comparable to those of atosiban. Whether such effects do also occur in primary cultures of myometrial cells deserves further investigations. Using the myograph model, we previously showed that BPJ concentration-dependently inhibits spontaneous myometrium contractions, affecting their peak, tension, and duration (Fürer et al., 2016; Santos et al., 2019a). Stronger effects on spontaneous contractility were observed when BPJ was combined with atosiban (Santos et al., 2019b). However, a comparison of findings in these models should be done with caution given that the modulation of contractility in myometrium strips is a much more complex process than what can be investigated with a cell line.

Flavonoid glycosides are known to be hydrolyzed by gut microbiota, leading to release of aglycons (Braune and Blaut, 2016; Cassidy and Minihane, 2017). In our experimental settings, the effects of FEF and A-Mix were comparable, suggesting that cleavage of the sugar moieties does not affect their activity in the models used. Only in the combination experiments with BEF, A-Mix had weaker effects than FEF. It may be that the glycosides contribute to a stronger synergistic effect, or that FEF contains additional compounds that are relevant for the activity.

Preterm birth is often associated with increased myometrium contractility, but uterus inflammation is also an important risk factor for fetal and neonatal central nervous system damage (Romero et al., 2014). Therefore, an ideal tocolytic agent should have both functions. Our results show that BPJ, and in particular the corresponding bufadienolides, prevent the OT-induced phosphorylation of two relevant MAPKs, namely SAPK/JNK and ERK1/2. This suggests that downstream enzymes might also be inhibited and prostaglandin production lowered as a consequence. Whether *B. pinnatum* preparations can inhibit inflammatory processes that might lead to increased myometrial contraction and eventually parturition needs further investigation.

In the development of new tocolytic agents, a simultaneous inhibition of the immediate, calcium mediated canonical pathway and of the activation of MAPK-dependent pathways in the myometrium is nowadays seen as a required pharmacological profile (Kim et al., 2017). The present data show that *B. pinnatum* matches with these requirements, which in turn further substantiates its use in the treatment of preterm labor.

## Data Availability

The raw data supporting the conclusions of this article will be made available by the authors, without undue reservation

## Abbreviations

A-Mix, flavonoid aglycon mix; BEF, Bufadienolide-enriched fraction; ; BO, bersaldegenin-1,3,5-orthoacetate; BPJ, *Bryophyllum pinnatum* press juice; CI, Combination index; ERK1/2, extracellular signal-regulated kinases 1 and 2; FEF, Flavonoide-enriched fraction; hTERT, Human myometrial telomerase reverse transcriptase cells; IP_3_, Inositol-1,4,5-trisphosphate; MAPK, Mitogen activated protein kinase; OT, Oxytocin; OTR, Oxytocin receptor; PHM1-41, Pregnant human myometrial cells; *p*-ERK1/2, Phosphorylated form of ERK1/2; p-p38, Phosphorylated form of p38; *p*-SAPK/JNK, Phosphorylated form of SAPK/JNK; SAPK/JNK, stress-activated protein kinase/c-Jun amino-terminal kinases (Ca^2+^), Calcium ions [Ca^2+^]_i_, Intracellular Calcium concentration; Δ[Ca^2+^]_i_, variation of intracellular Calcium concentration
