# Corrigendum: *Bryophyllum pinnatum* compounds inhibit oxytocin-induced signaling pathways in human myometrial cells

**DOI:** 10.3389/fphar.2023.1141346

**Published:** 2023-02-08

**Authors:** Stefanie Santos, Leonie Zurfluh, Mónica Mennet, Olivier Potterat, Ursula von Mandach, Matthias Hamburger, Ana Paula Simões-Wüst

**Affiliations:** ^1^ Department of Obstetrics, University Hospital Zürich (USZ), University of Zurich (UZH), Zurich, Switzerland; ^2^ Division of Pharmaceutical Biology, University of Basel, Basel, Switzerland; ^3^ Weleda AG, Arlesheim, Switzerland

**Keywords:** Bryophyllum pinnatum, Kalanchoe pinnata, myometrium cells, oxytocin, MAPK, intracellular calcium, cell signaling

In the published article, there was an error for the units used to report the press juice concentration. The units were incorrectly written as µg/mL, instead of mg/mL. In the original article, this error appeared in the legend for Figure 4, in Figures 1–3 and in the **Materials and Methods and Results** sections.

A correction has been made to **Materials and Methods**, Phosphorylation Experiments, Paragraph number 1. This sentence previously stated:

“hTERT-C3 cells (4.7 × 10^4^ cell/mL) were seeded into 6-well plates three to 4 days before experiments were performed. Once 90% confluence was reached, cells were treated with OT (100 nM) for 2, 5, 15, 30 or 45 min. To investigate the modulation of OT-induced phosphorylation under these conditions, cells were pre-treated with BPJ (2% corresponding to 20 μg/mL), atosiban (100 nM) or just medium. In additional experiments, cells were pre-treated with BPJ (20 μg/mL), BEF (2.20 μg/mL), FEF (17.39 μg/mL), A-Mix (0.68 μg/mL), BEF plus FEF and BEF plus A-Mix (same concentrations as in the single treatments), or just medium for 30 min before stimulation with OT for 5 min.”

The corrected sentence appears below:

“hTERT-C3 cells (4.7 × 10^4^ cell/mL) were seeded into 6-well plates three to 4 days before experiments were performed. Once 90% confluence was reached, cells were treated with OT (100 nM) for 2, 5, 15, 30 or 45 min. To investigate the modulation of OT-induced phosphorylation under these conditions, cells were pre-treated with BPJ (2% corresponding to 20 mg/mL), atosiban (100 nM) or just medium. In additional experiments, cells were pre-treated with BPJ (20 mg/mL), BEF (2.20 μg/mL), FEF (17.39 μg/mL), A-Mix (0.68 μg/mL), BEF plus FEF and BEF plus A-Mix (same concentrations as in the single treatments), or just medium for 30 min before stimulation with OT for 5 min.”

The corrected Figure 1, Figure 2 and Figure 3 and the corresponding captions appear below.

**FIGURE 1:**
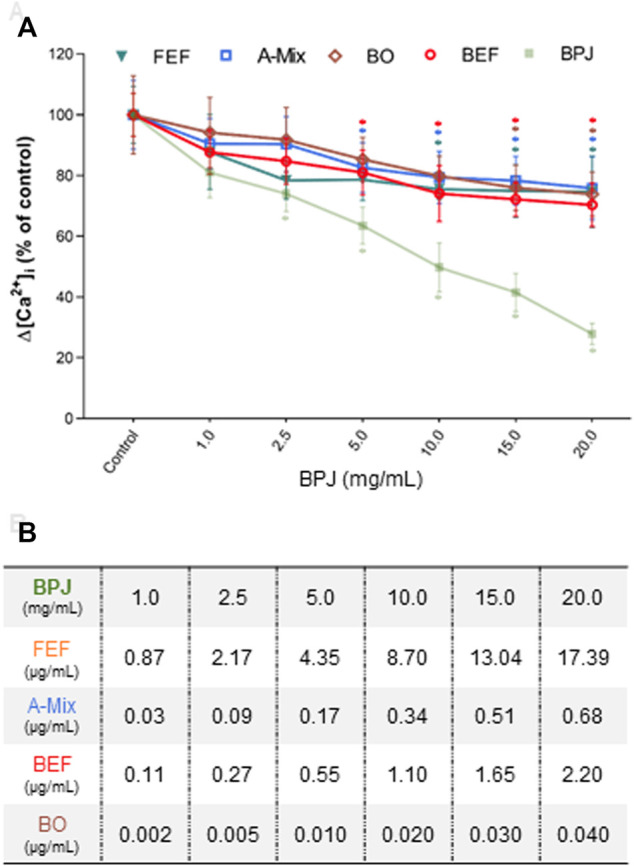
Concentration-dependent effects of BPJ fractions/compounds on the OT-induced increase of [Ca^2+^]_i._ Myometrial hTERT-C3 cells were pre-treated with BPJ, FEF, A-Mix, BEF or BO prior to stimulation with 100 nM of OT **(A)**. Values represent the mean ± SEM of six independent experiments performed in quadruplicate and are expressed as percentage of control; *p < 0.05. In **(B)**, the concentrations of FEF, BEF, A-Mix and BO corresponding to the BPJ concentrations are shown.

**FIGURE 2:**
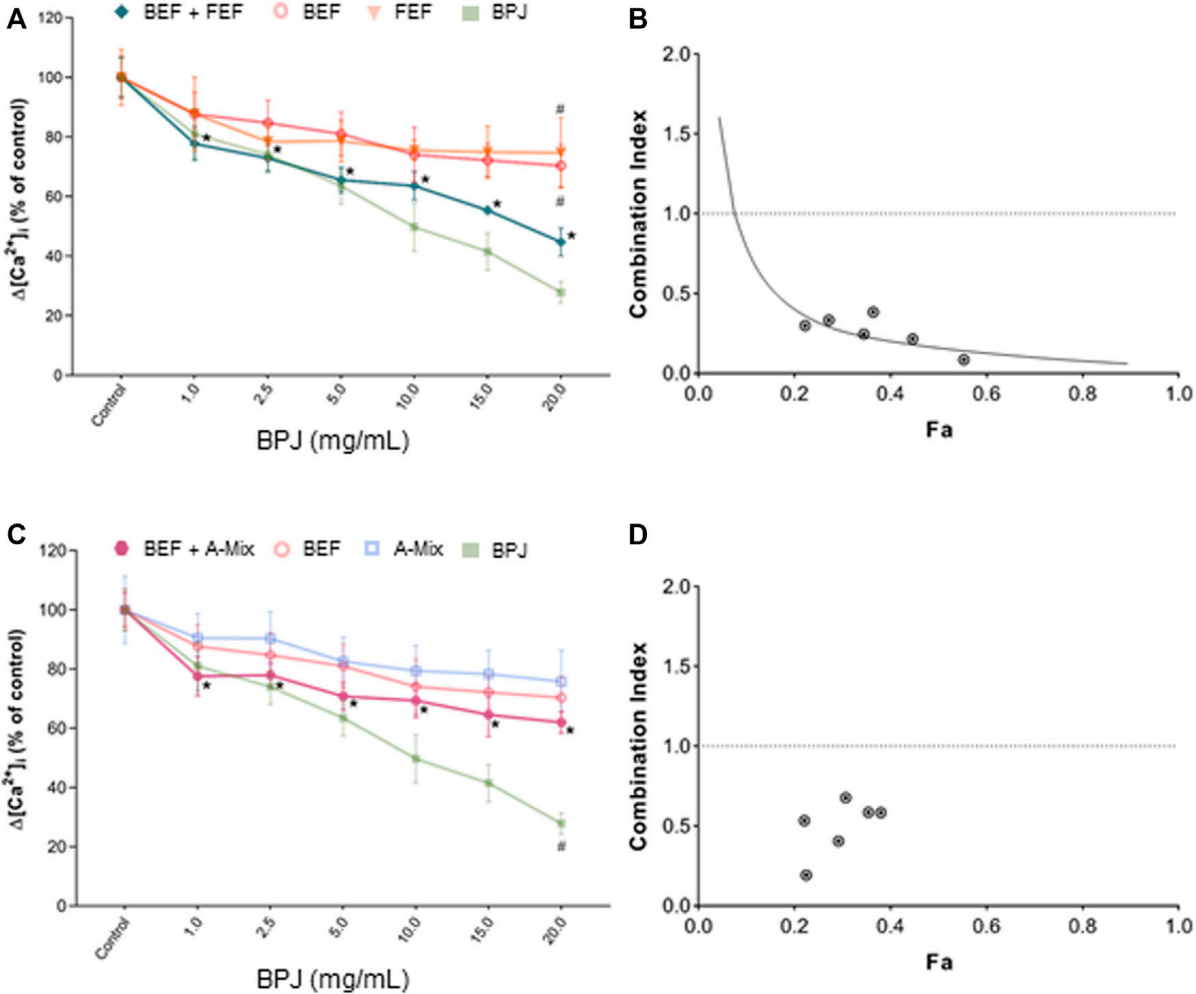
Effect of the combination of BPJ fractions/compounds on the OT-induced rise of [Ca^2+^]_i_. Cells were pre-treated with BEF plus FEF **(A)**, or with BEF plus A-Mix **(C)** prior to stimulation with 100 nM OT. Results of pre-treatment with BPJ and each substance alone are shown in transparent lines (significance symbols regarding comparison to control omitted). Values represent the mean ± SEM of six independent experiments performed in quadruplicate and are expressed as percentage of control; *p < 0.05 compared with control; ^#^ *p* < 0.05 compared to combination. Combination index (CI) values of the combination of BEF with FEF **(B)** or with A-Mix **(C)** were calculated from the concentration-response curves. Data were analyzed by the median-effect method. Fa: fraction affected.

**FIGURE 3:**
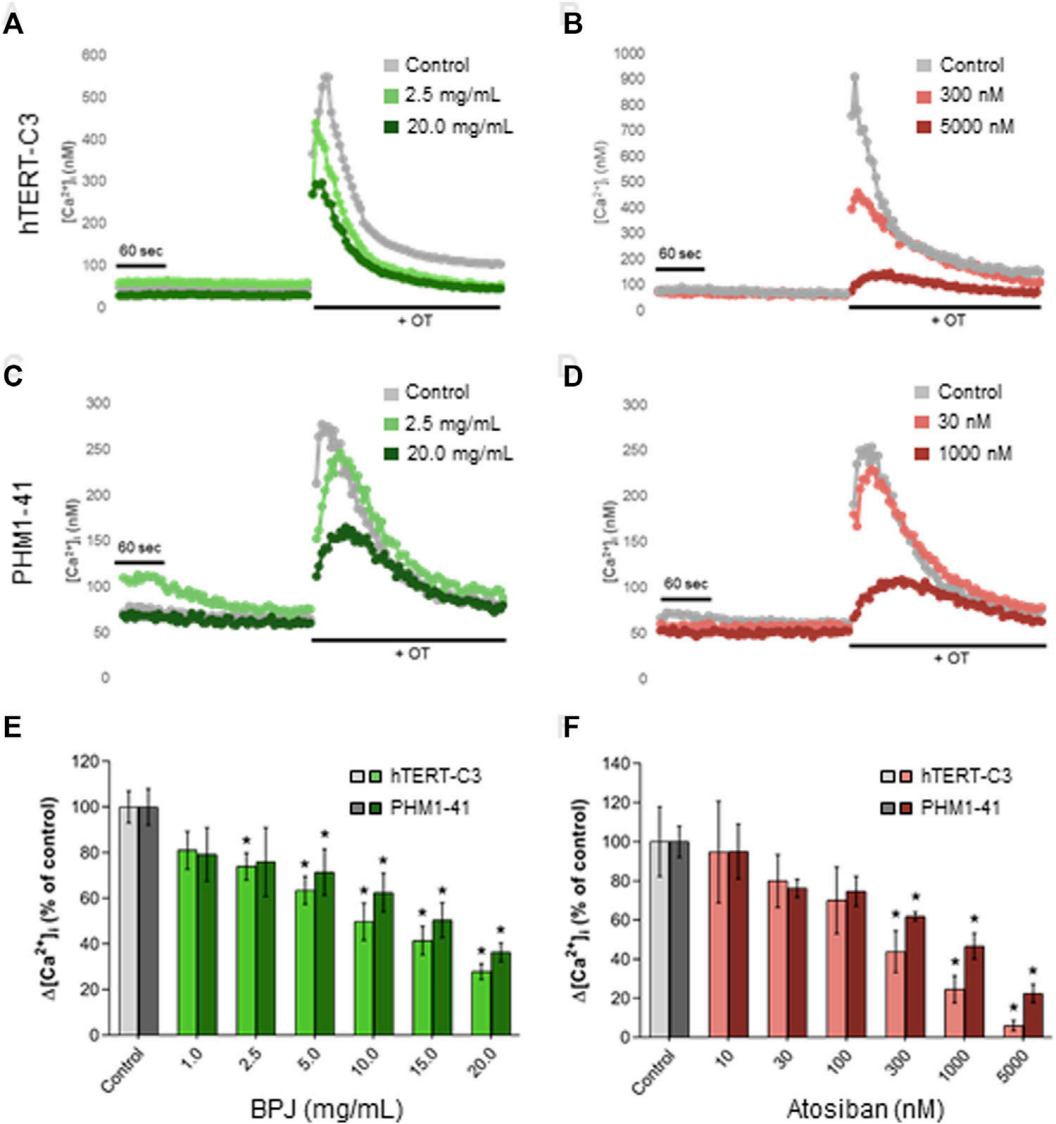
Comparison between the effects of BPJ and atosiban on OT-induced rise of [Ca^2+^]_i_ in myometrial cell lines. Time-course of OT-induced [Ca^2+^]_i_ response in hTERT-C3 **(A, B)** and PHM1-41 **(C, D)** cells when pre-incubated in the absence (**a-d**, grey lines) or in the presence of BPJ **(A, C)** or atosiban **(B, D)** [Ca^2+^]_i_ was measured for 4 min before stimulation with 100 nM OT. Data shown are from one representative experiment. Concentration-dependent effect of BPJ **(E)** or atosiban **(F)** on the oxytocin-induced [Ca^2+^]_i_ increase in hTERT-C3 (lighter color) and PHM1-41 (darker color) cells. Values represent the mean ± SEM of 4 (PHM1-41) or 6 (hTERT-C3) independent experiments performed in quadruplicate and are expressed as percentage of control; *p < 0.05.

The corrected Figure 4 caption appears below.

“FIGURE 4 Effect of BPJ fractions/compounds and atosiban on OT-induced MAPKs phosphorylation. In the time-course experiments, hTERT-C3 cells were pre-treated with or without 20 mg/mL BPJ or 100 nM atosiban for 30 min, before incubation with 100 nM OT for 2, 5, 15, 30, and 45 min **(A, C, E)**. To compare the effects of BPJ and the various fractions/compounds, cells were pre-treated with 20 mg/mL BPJ, 2.20 μg/mL BEF, 17.39 μg/mL FEF, 0.68 μg/mL A-Mix, BEF plus FEF, or BEF plus A-Mix (same concentrations as for single fractions) for 30 min before stimulation with OT for 5 min **(B, D, F)**. Whole cell proteins were subjected to western blot analysis with antibodies against phosphorylated p38 **(A, B)**, SAPK/JNK **(C, D)** and ERK1/2 **(E, F)**; matching densitometry analyses are depicted bellow the representative blots. Samples from the same experiment were processed in parallel and membranes were probed with GAPDH to confirm equal loading. Blot images are from a representative experiment, and line between bands delineate boundary between the gels that were cropped. Values represent the mean ± SEM of four independent experiments; *p < 0.05 compared with control; ^#^ *p* < 0.05 compared to OT-treated.”

A correction has been made to **Results**, BPJ inhibits OT-induced rise of [Ca2+]i in PHM1-41 myometrial cells, Paragraph number 1. These sentences previously stated:

“As previously shown, the exposure of hTERT-C3 cells to 100 nM OT induced an increase of [Ca^2+^]_i_ with a peak response at about 10–20 s after stimulation, and a subsequent decrease to resting conditions (Figures 3A,B). Pre-incubation with BPJ (0.1%–2.0% corresponding to 1.0–20.0 μg/mL) led to a concentration dependent decrease of the [Ca^2+^]_i_ peak induced by OT (*p* < 0.0001; Figure 3A). To verify if this effect was cell line dependent, experiments were conducted in a second myometrial cell line (PHM1-41). A peak response of [Ca^2+^]_i_ was observed at 12–20 s after stimulation with OT (Figures 3C,D). When pre-incubated with BPJ, a decrease of the OT-induced increase of cytosolic [Ca^2+^]_i_ peak was observed (Figure 3C). BPJ thus promoted a concentration-dependent effect on Δ[Ca^2+^]_i_ in both cell lines (*p* < 0.0001; Figure 3E), with significant effects at concentrations >2.5 μg/mL in hTERT-C3 cells (*p* = 0.012), and >5.0 μg/mL in PHM1-41 cells (*p* = 0.026).”

The corrected sentence appears below:

“As previously shown, the exposure of hTERT-C3 cells to 100 nM OT induced an increase of [Ca^2+^]_i_ with a peak response at about 10–20 s after stimulation, and a subsequent decrease to resting conditions (Figures 3A,B). Pre-incubation with BPJ (0.1%–2.0% corresponding to 1.0–20.0 mg/mL) led to a concentration dependent decrease of the [Ca^2+^]_i_ peak induced by OT (*p* < 0.0001; Figure 3A). To verify if this effect was cell line dependent, experiments were conducted in a second myometrial cell line (PHM1-41). A peak response of [Ca^2+^]_i_ was observed at 12–20 s after stimulation with OT (Figures 3C,D). When pre-incubated with BPJ, a decrease of the OT-induced increase of cytosolic [Ca^2+^]_i_ peak was observed (Figure 3C). BPJ thus promoted a concentration-dependent effect on Δ[Ca^2+^]_i_ in both cell lines (*p* < 0.0001; Figure 3E), with significant effects at concentrations >2.5 mg/mL in hTERT-C3 cells (*p* = 0.012), and >5.0 mg/mL in PHM1-41 cells (*p* = 0.026).”

The authors apologize for this error and state that this does not change the scientific conclusions of the article in any way. The original article has been updated.

